# Impact of early menarche on increased anxiety levels in female patients

**DOI:** 10.1192/j.eurpsy.2025.630

**Published:** 2025-08-26

**Authors:** K. Laskarin, E. Ivezic, E. Gudelj, S. Belcic, K. Matic, N. Ruljancic, V. Grosic, I. Filipcic, S. Vuk Pisk

**Affiliations:** 1 University Psychiatric Hospital Sveti Ivan, Zagreb; 2 Faculty of Dental medicine and Health Osijek, Osijek; 3 School of Medicine University of Zagreb, Zagreb, Croatia

## Abstract

**Introduction:**

Previous studies show contradictory results about the relationship between the age of menarche and the intensity of anxiety symptoms. Some studies found that anxiety symptoms were significantly higher in patients with earlier age of onset of menarche. Recent studies show that early puberty and menarche are associated with greater rates of morbidity of anxiety and other psychiatric illnesses than relatively late menarche. It is presumed that girls who achieve menarche earlier are less prepared for puberty and tend to have more negative emotions associated with menstruation.

**Objectives:**

The purpose of this research was to determine the correlation between onset of menarche and intensity symptoms of anxiety in female patient with affective and anxiety disorders.

**Methods:**

The research is prospective and includes female patients with established diagnoses of depressive disorder, anxiety-depressive disorder, bipolar disorder (depressive episode) aged 18-65. The patients had their laboratory parameters determined, including sex hormones (estrogen, progesterone, testosterone, FSH, LH and prolactin), filled out a demographic questionnaire and questionnaires: The Suicide Behaviors Questionnaire-Revised (SBQ-R), Generalised Anxiety Disorder Assessment (GAD-7), Patient Health Questionnaire (PHQ-9), Beck Depression Inventory (BDI-II), Beck Anxiety Inventory (BAI), Matthey Generic Mood Question. Montgomery-Asberg Depression Rating Scale (MADRS), Hamilton Anxiety Rating Scale (HAMA) i Hamilton Depression Rating Scale (HAMD).

**Results:**

The preliminary data of the prospective study showed that there was a statistically significant proportion of patients in whom a correlation was found between the age of onset of menarche and the intensity of anxiety.

**Conclusions:**

Age of menarche could be an influence on intensity of anxiety symptoms in female patients.

**Disclosure of Interest:**

None Declared

